# Coupling Cell Size Regulation and Proliferation Dynamics of *C. glutamicum* Reveals Cell Division Based on Surface Area

**DOI:** 10.1101/2023.12.26.573217

**Published:** 2023-12-28

**Authors:** César Nieto, Sarah Täuber, Luisa Blöbaum, Zahra Vahdat, Alexander Grünberger, Abhyudai Singh

**Affiliations:** a Department of Electrical and Computing Engineering, University of Delaware. Newark, DE 19716, USA; b CeBiTec, Bielefeld University. Bielefeld, Germany.; c Multiscale Bioengineering, Technical Faculty, Bielefeld University. Bielefeld, Germany.; d Institute of Process Engineering in Life Sciences: Microsystems in Bioprocess Engineering, Karlsruhe Institute of Technology. Karlsruhe, Germany.; e Center for Bioinformatics and Computational Biology, University of Delaware, Newark, DE 19716 USA.

## Abstract

Single cells actively coordinate growth and division to regulate their size, yet how this size homeostasis at the single-cell level propagates over multiple generations to impact clonal expansion remains fundamentally unexplored. Classical *timer* models for cell proliferation (where the duration of the cell cycle is an independent variable) predict that the stochastic variation in colony size will increase monotonically over time. In stark contrast, implementing size control according to *adder* strategy (where on average a fixed size added from cell birth to division) leads to colony size variations that eventually decay to zero. While these results assume a fixed size of the colony-initiating progenitor cell, further analysis reveals that the magnitude of the intercolony variation in population number is sensitive to heterogeneity in the initial cell size. We validate these predictions by tracking the growth of isogenic microcolonies of *Corynebacterium glutamicum* in microfluidic chambers. Approximating their cell shape to a capsule, we observe that the degree of random variability in cell size is different depending on whether the cell size is quantified as per length, surface area, or volume, but size control remains an adder regardless of these size metrics. A comparison of the observed variability in the colony population with the predictions suggests that proliferation matches better with a cell division based on the cell surface. In summary, our integrated mathematical-experimental approach bridges the paradigms of single-cell size regulation and clonal expansion at the population levels. This innovative approach provides elucidation of the mechanisms of size homeostasis from the stochastic dynamics of colony size for rod-shaped microbes.

## Introduction

Cell growth and cell cycle duration are interconnected variables. They determine not only the distribution of cell sizes but also the dynamics of colony expansion [1–3]. Although interdependent, cell size and cell population dynamics have been explored almost independently. From the perspective of cell size, recent research has revealed that most cells that grow exponentially control their division and therefore their proliferation employing the *adder* strategy, where cells add, on average, a fixed size from cell birth to division [4]. Although predictions on cell size statistics have been well documented [5–9], only a few details on the effects on population expansion have been studied [10–13]. These efforts mostly focus on analyzing average quantities, such as the mean growth rate or the cycle duration, with minimal consideration of its stochastic properties.

Cell proliferation is known to show appreciable random variability (noise) from colony to colony [1, 14]. The degree of variability in the number of individuals in different colonies has notable consequences from an ecological perspective [15]. For example, noise in cell proliferation can allow the fixation of a mutant strain in competition with others, even without showing a fitness advantage [16,17]. Furthermore, analyzing the growth dynamics of the microbial population can reveal details of pathogen infection in the host [18, 19]. This kind of analysis can be applied to other processes, such as the prevalence rate of a phenotype in fluctuation tests [20,21].

A detailed model describing colony expansion must be coupled with cell size control variables such as cell size growth and cell cycle duration. A better understanding of these variables has been achieved with the development of microfluidic techniques that allow tracking of thousands of cells over tens of hours [22, 23]. One of the most popular microfluidic devices is the *mother machine*, where a rod-shaped cell (the *mother*) is trapped in a closed channel and all its descendant cells are discarded [24, 25]. Tracking the growth and division statistics of these *mother* cells unveiled the *adder* strategy [26].

The origin of the *adder* is still unclear, since cell division involves multiple mechanisms [27]. Among them, we can highlight septum ring formation [6, 28], DNA replication [29], and cell wall synthesis [30, 31] that have to reach a high synchronization in a complex way. Recent studies simplify the division process to obtain simple conclusions by exploring the link between cell division and cell geometry [5, 32, 33]. These models can predict the dynamics of the cell size distribution with high precision [8], and can also predict weak damping oscillations in the statistical moments of the size distribution [7, 8, 34, 35]. However, it is not clear whether these oscillations can also affect the dynamics of colony growth.

This article aims to understand how cell division and colony proliferation are related to the geometric properties of cell shape. We focus on the regulation of cell size of *Corynebacterium glutamicum* cells, which have a rod-shaped morphology and grow limited by cell wall synthesis [36, 37]. Given these constraints, we expect to find differences in the analysis of cell proliferation using different cell size proxies, such as cell length, volume, and surface area. These differences can be explored by using our microfluidic device, which allows cells to grow with fewer shape restrictions than the *mother machine* [38, 39]. Moreover, since descendant cells remain in the chambers for many generations, it allows us to study the dynamics of colony population.

The article is structured as follows: In Section 1, we explore how the *adder* division strategy affects colony proliferation in bacteria. We compare it with the *timer* strategy, where cells divide after a fixed time. In Section 2, we present our experimental setup and preliminary results of the colony population variability. In Section 3, we measure cell length, surface area, and volume, and study their variability. We find that different cell size proxies yield different distributions. Finally, in Section 4, we analyze the variability in cell number over time for synchronized colonies. We compare models based on each of these cell proxies (length, surface, and volume) and discuss which of them aligns better with the observed proliferation dynamics.

## Cell proliferation strategies: *timer* versus *adder*

In this section, we compare the dynamics of cell population noise for the *adder* and *timer* division strategies. The *timer*, a simple and widely used model of population dynamics, assumes that cells divide on average after a fixed time [15, 40, 41]. The *adder* considers that the duration of the cell cycle is related to the cell size [5, 32]. First, we will describe how to study the statistics of the colony population. Then, we present the results for the models using a simple rate-based perspective and show how they differ in their predictions of population noise. Finally, we explain how to modify these simple models to better compare them with the experimental data.

### Quantification of the noise in population numbers

**Figure 1:**
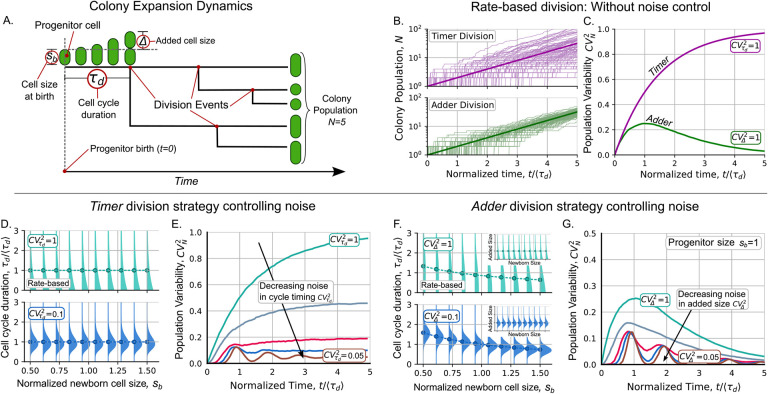
The *adder* division strategy is more effective in controlling the stochastic variation in colony number compared to the *timer* division strategy. **A.** Main variables in the colony expansion dynamics. **B.** Examples of the colony population dynamics for *timer* (top pink) and *adder* division (bottom green). **C.** Variability of the colony population over time for the two division strategies in their simplest approach. **D.** In the *timer* model, the cycle duration *τ*_*d*_ is independent and its variability is given by $CV_{\tau_{d}}^{2}$. **E.** Dynamics of the variability of the colony population for $CV_{\tau_{d}}^{2}\in \{1,0.5,0.2,0.1,0.05\}$. **F.** In the *adder* model, the distribution of *τ*_*d*_ depends on the size at the beginning of the cell cycle *s*_*b*_ through the added size Δ in eq. (4). The mean *τ*_*d*_ given *s*_*b*_ (dots) decreases with *s*_*b*_. *Inset:* the mean added size 〈Δ〉 is independent of *s*_*b*_. **G.** Dynamics of $CV_{N}^{2}$ for $CV_{\Delta }^{2}\in \{1,0.5,0.2,0.1,0.05\}$. While for the *timer $CV_{N}^{2}$* increases to a constant value, for the *adder*, $CV_{N}^{2}$ reaches its maximum around the first division and then decreases to zero. **Parameters**: For *timer*, *τ*_*d*_ is distributed by gamma with the specified 〈*τ*_*d*_〉 and $CV_{\tau_{d}}^{2}$. For *adder*, *s*_*b*_ = 〈Δ〉 = 1 and Δ is distributed by gamma with the specified $CV_{\Delta }^{2},\mu =ln\;(2)$.

To study the dynamics of proliferation, we initiate each colony with a single progenitor cell. This progenitor is a newly born cell of size *s*_*b*_ (see Figure 1A). We define the beginning of colony growth as the instant of progenitor birth (*t* = 0). Let *τ*_*d*_ be the random variable that defines the duration of the cell cycle, that is, the time between two consecutive divisions. After completing its cell cycle, this progenitor will split into two cells that will also divide and proliferate. If 〈.〉 corresponds to the average operator, *τ*_*d*_ has a mean of 〈*τ*_*d*_〉, known as the doubling time [42]. Since *τ*_*d*_ is a stochastic variable, the colony population or the number of individuals in the colony, denoted by *N*, is also noisy. To quantify how the division strategy affects the noise in *N*, we calculate the dynamics of the statistical moments of *N* estimated over different independent colonies. The first moment 〈*N*〉 corresponds to the mean colony population. The variability of the population is quantified using the squared coefficient of variation $CV_{N}^{2}\;:=\;\sigma_{N}^{2}/\langle N\rangle^{2}$, where $\sigma_{N}^{2}$ is the variance of *N*. A summary of the symbols used throughout this manuscript is provided in Table 1.

### Rate-based division models: The simplest approximation

From a theoretical perspective, it is common to define division as a stochastic jump process that occurs at a given rate [43,44]. A rate-based model considers the division to occur with a given propensity similarly to chemical reactions. For the *timer* strategy, division occurs at a constant rate 1*/*〈*τ*_*d*_〉. Thus, the division times follow exponential distribution with the property that the noise in the cell cycle time satisfies $CV_{\tau_{d}}^{2}:=\sigma_{\tau_{d}}^{2}/{\left\langle \tau_{d}\right\rangle }^{2}=1$ independently of 〈*τ*_*d*_〉. In the supplementary information, show that the distribution of population number is geometric with mean 〈*N*(*t*)〉 = exp(*μt*) with *μ* = ln(2)*/*〈*τ*_*d*_〉. Consequently, the population variability increases over time as follows:

(1) $${\left(CV_{N}^{2}\right)}_{\text{timer}}=1-e^{-\mu t}.$$

Some examples of population trajectories with division occurring at this constant rate are shown in Figure 1B. On the other hand, Figure 1C shows how the noise reaches 1 as time increases.

For the *adder* division strategy, the division rate is proportional to the cell size [45]. This strategy considers that the cell size *s* is growing exponentially at a rate *μ*:

(2) $$\frac{ds}{dt}=\mu s.$$

The cell size at the beginning of the cycle *s*_*b*_, also known as cell size at birth, is related to the size at division *s*_*d*_, through the added size during the cell cycle Δ = *s*_*d*_ *− s*_*b*_. By solving Eq. (2), we can relate these variables to the cell cycle duration *τ*_*d*_, as:

(3) $$s_{d}=s_{b}e^{\mu \tau_{d}}=s_{b}+\Delta \text{.}$$

In the supplementary information, we show that, using a rate proportional to cell size, Δ is predicted to be a random variable with exponential distribution $\left(CV_{\Delta }^{2}=1\right)$ as shown in Figure 1F. This means that *τ*_*d*_ is not an independent variable as in the *timer* but it depends on *s*_*b*_ through Δ following the formula obtained from Eq. (3):

(4) $${\left(\tau_{d}\right)}_{\text{adder}}=\frac{1}{\mu }ln\;\left(\frac{s_{d}}{s_{b}}\right)=\left(\frac{1}{\mu }\right)\;ln\;\left(\frac{s_{b}+\Delta }{s_{b}}\right).$$

In Figure 1F, we show how the distribution of *τ*_*d*_ depends on *s*_*b*_. This dependence affects the dynamics of the variability of the colony population $CV_{N}^{2}$. Figure 1B shows how $CV_{N}^{2}$ reaches its highest value around the first division, then gradually decreases over time until it approaches zero. For a detailed approach, we previously proposed numerical methods to estimate $CV_{N}^{2}$ [1].

### Population noise is more effectively buffered by the *adder* strategy than by the *timer* strategy

On the other hand, Figure 1G shows the trends of $CV_{N}^{2}$ considering the *adder* strategy with controlled $\Delta \left(CV_{\Delta }^{2}<1\right)$. Biologically, this level of control can be increased considering multiple stages before division [32, 46]. Similarly to the rate-based model, for this controlled added size, $CV_{N}^{2}$ also converges asymptotically to zero. When the added size has a noise within the typical biological range, such as $CV_{\Delta }^{2}<0.2$ [25], $CV_{N}^{2}$ exhibits fluctuations over time before converging to zero.

### Heterogeneity in progenitor cell size increases variability in colony proliferation

**Figure 2:**
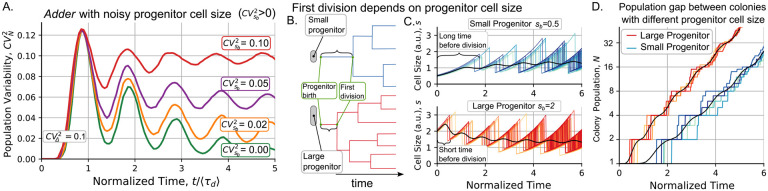
At increasing the noise in the progenitor size, the population variability does not decreases to zero but oscillates around a positive value. **A.** Dynamics of variability in the colony population $CV_{N}^{2}$ as increasing the noise in the progenitor cell size with $V_{s_{b}}^{2}\in \{0,0.02,0.05,0.1\}$. The larger the $CV_{s_{b}}^{2}$, higher the *b* the $CV_{N}^{2}$ asymptotic limit, and the lower the amplitude of the oscillations. **Parameters**: $\left\langle s_{b}\right\rangle =\langle \Delta \rangle =1,CV_{\Delta }^{2}=0.1$. **B.** Progenitor, the first cell of the colony born at *t* = 0, may have a random size. As the division rate is proportional to cell size, larger cells will divide earlier than smaller ones. **C.** Comparison of the dynamics of colonies derived from a small progenitor (*s*_*b*_ = 0.5〈Δ〉,blue shades) and a population from a large progenitor (*s*_*b*_ = 2〈Δ〉, red shades). Cell size over time and **D.** Colony population over time for different colonies. Different shades represent different simulated replicas. The black lines represent the mean population, which shows a permanent gap proportional to the difference of the progenitor cell size. Observe that the y-axis has logarithmic scale.

To plot Figure 1G, we simplify that the size of colony progenitor cell at *t* = 0 is *s*_*b*_ = 〈Δ〉 with probability one. In reality, *s*_*b*_ is a random variable with noise measured by the squared coefficient of variation $CV_{s_{b}}^{2}$. Figure 2A shows how by increasing $CV_{s_{b}}^{2}$ the asymptotic level of $CV_{N}^{2}$ also increases. From a mathematical perspective, this means that the steady properties of the system depend on its initial conditions.

To understand how population growth dynamics is affected by variability in progenitor size, it is important to examine how the timing of a progenitor’s first division is related to its size. Figure 2B illustrates that smaller progenitors generally experience a longer delay before their first division compared to larger progenitors. Figure 2D shows that the population expands asymptotically at an exponential rate after this first division. As a result, at a given time, colonies derived from small cells typically have a smaller population than those derived from large cells. This property, combined with the high control exerted by the division strategy on population number (Figure 1D), implies that the noise in the size of the progenitor cell is one of the main sources of variability in the population across colonies.

In our previous work [7,8], we showed how the *adder* strategy leads to oscillations in cell size moments, as observed in Figure 1C. We explained that these oscillations result from the synchronization of division and cell growth. Here, we show that this synchronization also affects the statistics of population number. Figure 1A shows that $CV_{N}^{2}$ oscillates and Figure 1D shows that the mean population follows a *staggered* growing instead of a smooth exponential growth. In the experiments explained in the next section, we will test how periodic these statistics are in practice.

## Experimental setup to study the *C. glutamicum* proliferation trends

To test the validity of the predictions of our model, we study the proliferation dynamics of the nonpathogenic gram-positive soil bacterium *C. glutamicum*. Originally isolated and used due to its natural ability to excrete L-glutamate [47], *C. glutamicum* is today used for the large-scale industrial production of various amino acids, particularly L-glutamate and L-lysine [48]. At the same time, *C. glutamicum* is a well-established model species for studies related to the cell wall in Corynebacteriales, including prominent human pathogens such as *Mycobacterium tuberculosis* and *Corynebacterium diphtheria*, because it shares the complex organization of the cell envelope with its pathogenic relatives [49].

**Figure 3:**
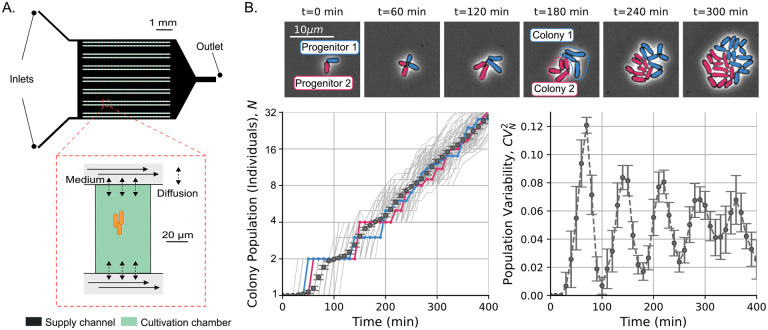
Experimental setup to investigate fluctuations in *C. glutamicum* colony sizes. **A.** The microfluidic single-cell cultivation (MSCC) device supplies a constant nutrient flow in 14 arrays of cultivation chambers using two inlets. **B.** (Top:) we show snapshots of the cell population for two colonies over time (*t*) each derived from a single progenitor. Different colors (blue and pink) represent different colonies. (Bottom-left:) Number of cells over time for 154 colonies (gray), with colored trajectories representing the colonies shown on top. Error-bars indicate the mean number of individuals over the studied colonies. (Bottom-right:) Noise in the colony population as the squared coefficient of variability over time (the error bars represent the 95% confidence interval for each statistical moment using bootstraping).

We used the microfluidic single-cell cultivation (MSCC) device [50, 51] to grow *C. glutamicum* cells. In this experimental setup, depicted in Figure 3A, cells can grow and proliferate for approximately 6 generations while we maintain controlled temperature and nutrient supply [52]. During this time, we captured snapshots of cell proliferation through phase contrast imaging. To segment cell contours and track lineages, we use DeLTa, an image processing software [53] followed by manual verification. Additional details on the experiments and the analysis workflow can be found in the Supplementary Information.

Next, we track the growth of cell colonies. As shown in Figure 3A, a colony consists of all descendants of a given progenitor cell, and colony growth begins at the progenitor birth. This theory requires synchronization of all colonies from the birth of their respective progenitor. This synchronization is generally hard to achieve experimentally and must be done when analyzing the data [8]. Figure 3B presents our preliminary results on how colonies proliferate over time. At first glance, the observed number of cells appears to grow similarly to the simulated data in Figure 2D. Similarly, $CV_{N}^{2}$ shows oscillations as predicted in Figure 2A. Next, we will conduct a more comprehensive investigation of these oscillations and their relationship to cell size regulation.

## Analysis of the geometric dimensions for *C. glutamicum* cells

The main conclusion from the previous sections is that the added size noise $CV_{\Delta }^{2}$ and the progenitor size noise $CV_{s_{b}}^{2}$ are the primary parameters to define the proliferation dynamics of a growing colony following the *adder* division strategy. It should be noted that the particular definition of $CV_{s_{b}}^{2}$ does not assume any specific geometric property for cell size. In this section, we examine how $CV_{s_{b}}^{2}$ differs if cell size is measured in terms of length, surface area, or volume.

### Approximating cell shape as a capsule

**Figure 4:**
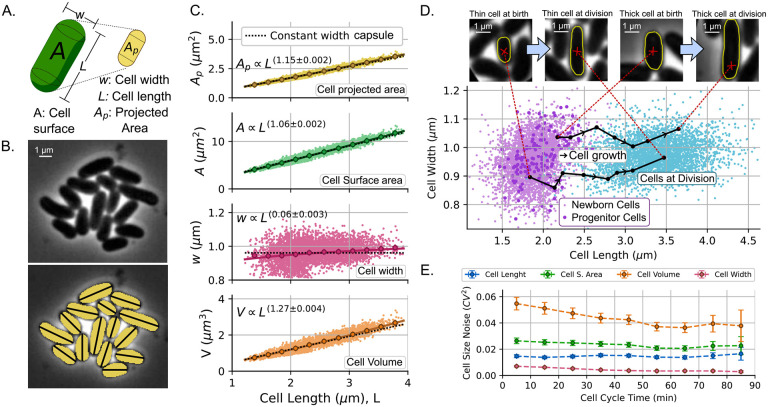
By assuming bacteria have a capsule shape, dimensions are not linear functions of their length. Therefore, noise is different for cell length, cell area, and cell volume. **A.** The cell shape is approximated to a capsule, with projected area *A*_*p*_ and length *L* measured from the images. The width *w*, volume *V*, and surface area *A* are estimated from *A*_*p*_ and *L*. **B.** A microscopic image example with cell segmentation (yellow pixels) and measured length (black lines). **C.** Experimental data (40891 datapoints) of projected area *A*_*p*_, cell surface *A*, cell width *w*, and cell volume *V* versus cell length *L* are compared to the approximation of the capsule shape with constant width and observed length (black dashed line). Power-law fitting (solid colored line) with the exponent is shown inset. **D.** Cell width versus cell length for newborn cells (violet) and cells at division (teal). On top, we present two cells (one thin and another thick) at the beginning and end of one cycle. The black lines show the cell dynamics along the cycle. **E.** Random variability of different cell dimensions, measured by the squared coefficient of variability as a function of the cycle time. Error bars represent the 95% confidence intervals for the statistics.

To simplify the cell geometry, we approximate the cells as capsules (Figure 4A), which consists of a cylinder and two semispheres. We estimate the cell dimensions from the images by segmenting the cell contours and measuring the projected area *A*_*p*_, which is proportional to the pixel count within the contour (Figure 4B). We define the cell length *L* as the longest side of the minimum-bounding rectangle of the contour. The projected area *A*_*p*_ and the cell length *L* are related to an effective cell width *w* by the formula of the projected area of a capsule:

(5) $$A_{p}=w\left(L-w\right)+\pi \;{\left(\frac{w}{2}\right)}^{2}.$$

Thus, from *A*_*p*_ and *L*, *w* is estimated by solving Eq. (5). To illustrate the precision of this approximation for *w*, we selected two cells (Figure 4D) with distinct and noticeable widths. The black lines in the plot width versus length in Figure 4D represent the width inference taken for the same cells at various points in their cell cycles until division. The fluctuations of these paths give us an idea of the error associated with the shape approximation Eq. (5) (approx 3%). Cell-to-cell variability (approx 15%) is greater than this inference error.

Once estimated *w*, the cell surface area *A* and volume *V* can be estimated as follows:

(6) $$A=\pi Lw;\;V=\pi (L-w)\;{\left(\frac{w}{2}\right)}^{2}+\frac{3}{4}\pi \;{\left(\frac{w}{2}\right)}^{3}=\frac{\pi Lw^{2}}{4}-\frac{\pi w^{3}}{16}.$$

Figure 4C illustrates how cell dimensions (*A*_*p*_, *A*, *w*, and *V*) scale with the length of the cell *L*. One notable observation regarding cell allometry (how cell dimensions scale with the length) is that area and volume do not scale perfectly linearly with cell length. Previous studies [25, 54] have assumed that cell size, area, and length scale linearly, supported by the low variability of cell width in confining microfluids such as the *mother machine*. However, our Supplementary Information shows that given the assumption of a capsule shape, non-linear allometric exponents emerge naturally even if the width is constant. In fact, closer observation of Figure 4C shows how the approximation of constant width fits relatively well with the best adjusted power laws (black dashed line). A more detailed approach about how a non-constant width can change the allometric exponent is presented in the Supplementary Information.

### Different proxies of cell size convey different statistics

The purpose of studying the geometric properties of cell shape is to study how variability in width and cell length affects the cell size noise. Consider that cells have a length with mean 〈*L*〉 and noise; $CV_{L}^{2}$; a width with 〈*w*〉 mean and noise $CV_{w}^{2}$. A dimension *F* (Area, volume, etc) calculated from *w* and *L* will have a noise approximated by:

(7) $$CV_{F}^{2}\approx CV_{w}^{2}\frac{\langle w\rangle^{2}}{\langle F\rangle^{2}}{\left(\frac{\partial F}{\partial w}\right)}_{w=\langle w\rangle }^{2}+CV_{L}^{2}\frac{\langle L\rangle^{2}}{\langle F\rangle^{2}}{\left(\frac{\partial F}{\partial L}\right)}_{L=\langle L\rangle }^{2}.$$

As an example of Eq. (7) for the surface area *F* = *A* = *πLw*, we have:

(8) $$CV_{A}^{2}\approx CV_{w}^{2}+CV_{L}^{2}.$$

If the cell volume is simplified from Eq. (6) to $F=V\approx \frac{\pi Lw^{2}}{4}$, we can approximate:

(9) $$CV_{V}^{2}\approx 4CV_{w}^{2}+CV_{L}^{2}.$$

From this result, we expect the volume to be a particularly noisy variable, since the contribution of the noise in width $CV_{w}^{2}$ is amplified by four. In the Supplementary Information, we quantify this noise amplification for the capsule shape using simulations finding that Eq. (9) is a reasonable approximation. Figure 4E shows the difference in noise for cell length, cell surface area, and cell volume at different times after their most recent division. We verify the approximations in Eq. (8) and Eq. (9) concluding that:

(10) $$CV_{L}^{2}<CV_{A}^{2}<CV_{V}^{2}.$$

A not intuitive property of this strain is that while the length and surface area maintain a constant *CV*^2^ throughout the cycle, the noise in cell volume decreases. In the Supplementary Information, we demonstrate that this decrease in volume noise is associated with the reduction of cell-width noise as cell length increases. The origin of this kind of regulation is still on debate [55].

This disparity in noises on cell size, especially among newborn cells (such as progenitors), is important because it defines the dynamics of population statistics, as explained before. To fit our simulations, we also measured the noise of the added size $\left(CV_{\Delta }^{2}\right)$. In the following section, we explore how accurate the assumption of *C. glutamicum* follows the *adder* strategy and we also measure $CV_{\Delta }^{2}$.

## Size regulation of *C. glutamicum* cells

**Figure 5:**
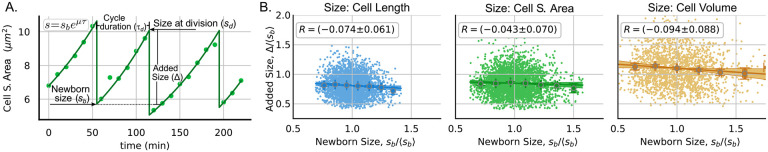
*C. glutamicum* cells control their size following the *adder* division strategy. **A.** An example of experimental trajectories of the cell surface over time (dots). The data were fitted to exponential functions of time (continuous line). The newborn size *s*_*b*_ and the size at division *s*_*d*_ are estimated by extrapolating the fitted exponential functions of time. Other parameters such as cycle duration and added size are also represented. **B.** Division strategy represented as the relationship between the added size Δ and the size at the beginning of the cell cycle *s*_*b*_ for 3345 cell cycles. This strategy was presented considering cell length (blue), cell surface area (green), and cell volume (orange). The statistics of Δ and *s*_*b*_ are presented in Table 1. The correlation coefficient *R* between Δ and *s*_*b*_ is also shown with a confidence interval error of 95 % calculated using bootstrap methods.

To model cell size regulation, an initial assumption is that cells grow exponentially over time. In Figure 5A, we compare the dynamics of the cell size observed with the approximation of exponential growth. Despite the ongoing debate on the nature of *C. glutamicum* size growth [56], the results demonstrate that, for the level in detail of this study, the exponential growth approximation is a suitable choice.

### *C. glutamicum* cells divide following the *adder*

Next, we examine whether the division of *C. glutamicum* follows the *adder* division strategy. To test this hypothesis, we plot the added cell size (Δ) against the birth size (*s*_*b*_) in Figure 5B. The main property of the *adder* strategy is that the added size, the difference between the size at division and the size at birth, is a variable with fixed mean independent of the size at birth [32]. This independence can be quantified by the Pearson correlation coefficient (*R*(Δ, *s*_*b*_)) between these variables. The results confirmed that *C. glutamicum* follows an *adder* division strategy since *R ≈* 0. To incorporate the *adder* into our simulation model, we treated the added size Δ as an independent random variable with the observed coefficient of variation $\left(CV_{\Delta }^{2}\right)$. Table 2 shows the measured $CV_{s_{b}}^{2}$ and $CV_{\Delta }^{2}$ that were used in our simulations.

## Bacterial proliferation statistics and comparison with predictions

**Figure 6:**
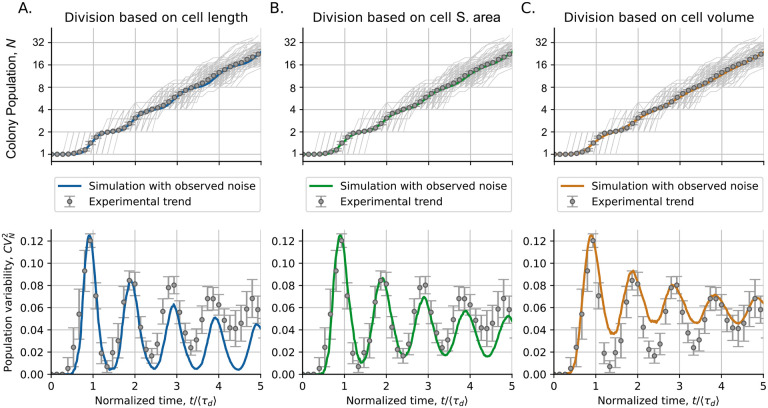
Division based on different proxies of cell size: length (A.), surface (B.), or volume (C.), predicts a similar mean population dynamics but different dynamics of population variability. The upper part of the figure shows the population in different colonies (gray lines) and their mean (error bars) compared to simulation predictions (colored lines). The bottom part shows the observed dynamics of population variability $CV_{N}^{2}$ (error bars) compared to the expected dynamics from the simulations (colored lines). Simulations were performed with observed noise in the size of the progenitor cell $CV_{s_{b}}^{2}$ and the size of the cell added before division $CV_{\Delta }^{2}$. Table 2 provides the exact value of these parameters. The error bars in experiments represent the 95% confidence interval on the moments over 154 studied colonies. The mean doubling time 〈*τ*_*d*_〉 is 75.2 ± 0.4 min.

To compare data and theory, we need to ensure that progenitor cells are newborns. We achieve this by synchronizing cell size trajectories a posteriori, so that all colony progenitors start their dynamics immediately after a division (see [8] for more details). We then count the number of cells derived from each progenitor and use it to plot Figure 6. To compare with theory, we performed simulations using our agent-based algorithm assuming that the progenitor cell size and the added cell size during the cell cycle follow gamma distributions with the observed statistics $CV_{s_{b}}^{2}$ and $CV_{\Delta }^{2}$ presented in Table 2. We provide more details of this algorithm in Supplementary Information.

### Cell surface area is the main contributor to the *C. glutamicum* division

Figure 6 presents the cell proliferation statistics for the colonies of *C. glutamicum*. The statistical moments of the population distributions are compared with the results of our individual-based simulations. Since cell size can be defined using three different cell variables (length, surface area, and volume), we compare the results of the simulations using each of these types of cell size. In Figure 6 top, we observe how the three models predict very accurate trends in the mean population 〈*N*〉. However, there is an appreciable difference in the prediction of trends of population variability $CV_{N}^{2}$ (Figure 6 bottom).

Cell length, the variable with relatively fewer fluctuations (less $CV_{\Delta }^{2}$ and $CV_{s_{b}}^{2}$) predicts the dynamics of population fluctuations with stronger oscillations and a lower value at the limit of *t →* ∞ (Figure 6A). The model based on the cell surface area predicts a trend in population variability with dynamics similar to that observed experimentally (Figure 6B). Finally, volume, the variable with higher variability, as explained by Eq. (10), predicts oscillations with a smaller amplitude and a higher value at the limit of *t →* ∞ (Figure 6C). The closer agreement between the observed proliferation fluctuations and the predictions of the model with division based on cell surface area, let us conclude that a molecular mechanism proportional to the cell surface area might be the main contributor to cell division.

## Discussion

In this article, we explore how different cell division strategies predict different statistics on the colony population number. Using a semi-analytical approach, we compare the *adder* strategy with the *timer* strategy. The *timer* is a widely used model to describe populations [21, 57–60] in which the timing of the cell cycle is independent of cell size. The *adder*, in contrast, does consider the cell size to define the probability of division. Understanding how these models describe the dynamics of the population in colonies of low numbers of cells is especially relevant when studying cell proliferation, such as tumors or infections [61–63]. We expect that the accuracy of these models can be improved by implementing a size-dependent division, as we explain here.

While the dynamics of noise in colony population for *timer* division can be defined by the variability of the cell cycle, in the *adder*, the dynamics is defined by two parameters: the variability of the progenitor cell size and the noise of the added size before division. The *timer* predicts a trend of population noise that increases monotonically over time until it reaches a constant value. On the other hand, the *adder* predicts oscillatory dynamics. The amplitude of these oscillations is modulated by the noise in added size: the lower the added size noise, the higher the amplitude of these oscillations. On the other hand, the noise in the progenitor cell size defines the level around which the noise in population number oscillates. In the case of noiseless progenitor size, the noise in the colony number first increases, reaching the maximum during the first division, and then it reaches zero eventually.

Intuitively, it is possible to explain why the *adder* controls accurately the cell proliferation compared to the *timer*. First, consider the total biomass *B*, defined as the sum of all cell sizes:

(11) $$B=\sum_{i=1}^{N}\;s_{i}=s_{b}e^{\mu t}.$$

Ignoring the noise in growth rate, for a given *s*_*b*_, in supplementary information, we show that $CV_{N}^{2}$ is related to the cell size noise $CV_{s}^{2}$ through:

(12) $$CV_{N}^{2}\overset{\langle N\rangle \to \infty }{⟶}\frac{1}{\langle N\rangle }CV_{s}^{2}.$$

If the cell division strategy leads to a relatively narrow cell size distribution, that is $CV_{s}^{2}<\infty$ as occurs for the *adder* [45], thus $CV_{N}^{2}$ will go to zero. In the case of the *timer* division, both $CV_{s}^{2}$ and 〈1*/N*〉 the diverge [64]. Therefore, Eq. (12) does not hold. In Supplementary Information, we show the details of this result. In simple words: *If the cell population is coupled with the total biomass through a division based on the cell size, the population number will grow exponentially with low variability, as does biomass*.

Our model does not assume a specific proxy for cell size (volume, surface area, or length). Therefore, if the noises of these dimensions can be distinguished significantly, we can test which dimension controls cell division by observing the population growth dynamics. Although our model is simple, it may reveal other hidden insights on the mechanisms of cell size regulation for other rod-shaped exponentially growing organisms, by analyzing the signatures of these oscillations. Further studies with different cell strains and growth conditions, together with genetic modifications, can help us to understand in a deeper way not only the details of cell proliferation, but also cell division, helping to address open questions in the field [65,66].

This article assumes some approximations to the complex physiology of cells. One of them is to simplify the shape of the cell to a capsule [67] although some cells may not have uniform widths or be slightly bent. This approximation may be necessary to obtain simple conclusions and the resolution of our images is not high enough to a more complex approach. Part of the main conclusion; that cell volume, surface and length have distinguishable noises, is a result of this capsule shape approximation. If more variable cell shapes are considered, we expect that the noise difference of these dimensions will be larger. Future experiments with higher-resolution images may facilitate a more detailed shape analysis.

We show that the *adder* strategy is a good approximation for the cell division strategy in *C. glutamicum*. Based on that division model, we use the method mentioned above to get the expected proliferation statistics. As the noise in progenitor size is different for the cell size proxies studied, the model predicts similar mean population trends but different trends in population noise. We find that the predicted dynamics of population noise fits better with the experiment if we assume that the division is defined by the cell surface area. Therefore, we suggest that this dimension of the cell, or a molecule with levels proportional to it, is the main contributor to cell division in this strain.

Our model can be further enriched by incorporating other variables that affect cell size regulation, such as the variability of growth rate or partitioning noise [68–70]. Although these variables may have a minor effect individually, together they may play a crucial role in improving the precision of cell proliferation predictions [7, 8]. However, the incorporation of all these variables can be complex due to the presence of hidden correlations [71]. For instance, recent studies have shown that correlations between cell growth rate and size at the beginning of the cell cycle [8, 72], and between the sizes of sibling cells at division [73].

Other hidden variables such as chromosome replication and replication initiation can also play a fundamental role [27]. Understanding how all these variables are correlated and contribute to the cell proliferation process requires a more theoretical approach [74]. Furthermore, genetic mechanisms can also affect cell proliferation by modifying the growth rate or cell shape [75–79]. Other possible effects to include may be changes in cell growth rate due to protein regulation [80], effects of other division strategies [81] and other variables that depend on initial conditions [82]. Although a more comprehensive model should include all these variables, we expect that the improvements of a more complex model will be minor and that the surface will still be the main determinant of cell division for this strain, since it is already known that the cell surface is the limiting factor for cell growth.

## Supplementary Material

Supplement 1

## Figures and Tables

**Table 1:** Symbols used in the text.

Variable	Interpretation
N	Cell population in a colony
$CV_{N}^{2}\;≔\;\frac{\sigma_{N}^{2}}{{\langle N\rangle }^{2}}$	Variability of the population across colonies.
$\tau_{d}$	Cell cycle duration.
$CV_{\tau_{d}}^{2}\;≔\;\frac{\sigma_{\tau_{d}}^{2}}{{\langle \tau_{d}\rangle }^{2}}$	Noise in the cycle duration.
s	Cell size (with a general metric).
μ	Exponential cell growth rate.
$s_{b}$	Newborn cell size (also progenitor).
$s_{d}\;≔\;s_{b}e^{\mu \tau_{d}}$	Size at division.
$\Delta \;≔\;s_{d}-s_{b}$	Added size before division.
$CV_{\Delta }^{2}\;≔\;\frac{\sigma_{\Delta }^{2}}{{\langle \Delta \rangle }^{2}}$	Noise in the added size before division.
$CV_{s_{b}}^{2}\;≔\;\frac{\sigma_{s_{b}}^{2}}{{\langle s_{b}\rangle }^{2}}$	Noise in the progenitor cell size.
V	Cell volume.
A	Cell surface area.
L	Cell length.
w	Cell effective width.
$A_{p}$	Projected cell area.

**Table 2:** Measured parameters of cell size regulation for *C. glutamicum*. We present the mean size of the progenitor cell 〈*s*_*b*_〉, its variability $CV_{s_{b}}^{2}$ the mean added size before division 〈Δ〉 and its variability $CV_{\Delta }^{2}$. The digit in parentheses represents the amount by which the least significant digit of the value is uncertain (95% confidence interval using bootstrap methods). For example, 0.1882(6) = (0.1882 ± 0.0006). 3345 cell cycles were studied.

Cell dimension	$\langle S_{b}\rangle$	$CV_{s_{b}}^{2}$	$\langle \Delta \rangle$	$CV_{\Delta }^{2}$
Length (*μ*m)	1.882(6)	0.0147(6)	1.526(8)	0.042(2)
S. Area (*μ*m^2^)	5.69(2)	0.025(1)	4.75(3)	0.058(2)
Volume (*μ*m^3^)	1.133(7)	0.052(2)	1.17(1)	0.093(4)

## Data Availability

Our Supplementary Information and scripts for data analysis and simulations can be found at: https://zenodo.org/records/10433707. DOI: 10.5281/zenodo.10433706.
