# Supplementary material for: Coupling Cell Size Regulation and Proliferation Dynamics of C. glutamicum Reveals Cell Division Based on Surface Area
Source: bioRxiv. 2023 Dec 28:2023.12.26.573217. Preprint. [Version 2] doi: 10.1101/2023.12.26.573217 (PMC10793411; doi:10.1101/2023.12.26.573217)
Supplement: Supplementary file 1 [file media-1.zip › Repository/SupplementaryInformation.pdf]

Supplementary Information: Coupling Cell Size Regulation and Proliferation Dynamics of *C. glutamicum* Reveals Cell Division Based on Surface Area

Rate-based division models

A mathematically simple approach to describe theoretically the division dynamics of growing cells is the stochastic hybrid systems. In this approach, the continuous exponential growth is accompanied with jumps meaning divisions that occurs at a particular rate. For the timer division, the division occurs at a constant rate $h = \mu$ and for the adder, this rate is $h = \frac{\mu}{\langle\Delta\rangle}s$ proportional to the instantaneous cell size $s = s_b e^{\mu\tau}$ where s_b is the size at the beginning of the cycle, τ is the time from the most recent division. To model a single division, given a general division rate $h(\tau)$ which is dependent on τ, we can start from the master equation:

$$\frac{dp_0}{d\tau} = -h(\tau)p_0$$
$$\frac{dp_1}{d\tau} = h(\tau)p_0 = h(1-p_1), \quad (1)$$

where p_0 is the probability that the cell has not been divided at time τ and p_1 is the probability that the cell divides. Observe that $p_0 + p_1 = 1$. The solution for p_1 in (1) is given by:

$$p_1(\tau) = 1 - \exp\left(-\int_0^\tau h(\tau')d\tau'\right). \quad (2)$$

The probability distribution function ρ_{τ_d} of the division time τ_d is found by using the property $\rho_{\tau_d}(\tau) = \frac{dp_1}{d\tau}$ as follows:

$$\rho_{\tau_d}(\tau) = h(\tau)exp\left(-\int_0^\tau h(\tau')d\tau'\right). \quad (3)$$

for the timer and adder cases, we obtain, respectively, the distributions:

$$(\rho_{\tau_d}(\tau))_{Timer} = \mu exp\left(-\mu\tau\right)$$
$$(\rho_{\tau_d}(\tau))_{Adder} = \mu\frac{s_b}{\langle\Delta\rangle}e^{\mu\tau}\exp\left(-\frac{s_b}{\langle\Delta\rangle}(e^{\mu\tau}-1)\right). \quad (4)$$

We can highlight that the distribution of the division times is exponential with mean $\langle\tau_d\rangle = 1/\mu$ and $CV^2_{\tau_d} = 1$ as we use in the main article.

On the other hand, the moments of τ_d for the adder division follow more complex expressions that depend on s_b. For simplicity, we can show that using the transformation $\Delta = s_b(e^{\mu\tau}-1)$, the distribution of the added size can be obtained from the distribution of τ_d [3].

$$\rho_\Delta(\Delta) = \frac{1}{\langle\Delta\rangle}\exp\left(-\frac{\Delta}{\langle\Delta\rangle}\right), \quad (5)$$

which, being also an exponential distribution with independence of other cycle variables, has noise $CV^2_\Delta = 1$.

Population distribution for a single-step timer division follows a geometric distribution

The simplest approximation to population dynamics corresponds to the Markovian process where each cells have a division rate $\mu = 1/\langle\tau_d\rangle$. If the population has N individuals, each with the same probability of get divided, the transition of colony population from N to $N+1$ will occur at a rate μN. This process can be described by the master equation:

$$\begin{aligned}
\frac{dp_1}{dt} &= -\mu p_1 \\
\frac{dp_2}{dt} &= -2\mu p_2 + \mu p_1 \\
&\vdots \\
\frac{dp_N}{dt} &= -N\mu p_N + \mu(N-1)p_{N-1} \\
&\vdots \quad , \qquad (6)
\end{aligned}$$

where $p_N(t) \in (0,1)$ is the probability that the colony has N individuals at time t. The integration of the system (6) can be done sequentially. Considering the initial condition: $p_1(0) = 1$, $p_N(0) = 0$ if $N > 1$, we obtain:

$$\begin{aligned}
p_1 &= \exp(-\mu t) \\
p_2 &= \mu\int_0^t e^{-2\mu(t-\tau)}p_1(\tau)d\tau = \exp(-\mu t)(1-\exp(-\mu t)) \\
&\vdots \\
p_N &= \mu(N-1)\int_0^t e^{-N\mu(t-\tau)}p_{N-1}(\tau)d\tau \\
&= \exp(-\mu t)\left(1-\exp(-\mu t)\right)^{N-1} \\
&\vdots \quad , \qquad (7)
\end{aligned}$$

corresponding to the geometric distribution with mean population amount $\langle N\rangle = \exp(\mu t)$. Using the representation of this distribution, we solve for the first and second moments of the colony population:

$$\begin{aligned}
\langle N\rangle &= e^{\mu t} \\
\langle N^2\rangle &= e^{\mu t}\left(2e^{\mu t}-1\right). \qquad (8)
\end{aligned}$$

Quantifying the population noise: $CV^2_N = \frac{\langle N^2\rangle}{\langle N\rangle^2} - 1$, we obtain the dynamics of the population noise for a *timer*-based division:

$$CV^2_N = 1 - e^{-\mu t}. \quad (9)$$

A similar calculation of the population moments considering a cell cycle noise smaller than the one for exponential distribution requires an approach from non-markovian perspective which is further than the purpose of this article.

Approximate relationship between CV^2_Δ and $CV^2_{s_b}$

It is possible relate the noises CV^2_Δ and $CV^2_{s_b}$. Following a method similar to [1], we consider the size at birth s_b, the size at division s_d and the added size during a cell cycle Δ. Assuming the cell size is perfectly halved during division this means $s_b = s_d/2$ and therefore, we have $\mathrm{var}(s_d) = 4\mathrm{var}(s_b)$.

On the other hand, considering the *adder* principle:

$$s_d = s_b + \Delta, \tag{10}$$

gives us important properites. For instance, matching the mean of (10), we obtain $\langle s_b\rangle = \langle\Delta\rangle$ and matching the variances dividing by the mean squared, we obtain:

$$CV^2_{s_b} \approx \frac{1}{3}CV^2_\Delta, \tag{11}$$

which, at comparing with the experiments, it is satisfied for the length and the surface. In the case of the volume, we have found $CV^2_{s_b} > \frac{1}{3}CV^2_\Delta$. This discrepancy can provide information on changes in shapes and density during division since, if the division is nor perfectly symmetric, the equality $\mathrm{var}(s_d) = 4\mathrm{var}(s_b)$ does not hold. Instead, if we assume that during division, s_d is multiplied by a random variable β with $\langle\beta\rangle = 1/2$ and a given noise $0 < CV^2_\beta < 1$ following a similar reasoning:

$$\langle s_b^2\rangle = \langle\beta^2 s_d^2\rangle = \langle\beta^2\rangle\langle s_d^2\rangle, \tag{12}$$

and the equality $var(s_d) = var(s_b) + var(\Delta)$, it is possible to derive the relationship:

$$CV^2_{s_b} = \frac{CV^2_\beta CV^2_\Delta + CV^2_\beta + CV^2_\Delta}{3 - CV^2_\beta} > \frac{CV^2_\Delta}{3}, \tag{13}$$

as observed consistently in all the three observed proxies of size.

Asymptotic behavior of CV^2_N

Here, we study the asymptotic limit of CV^2_N and how it can be related to CV^2_s. Let us start with the definition of the total colony biomass:

$$B = \sum_{i=1}^{N} s_i = s_b e^{\mu t} \tag{14}$$

We assume that the mean cell size $\langle s\rangle$ can be related to the arithmetic mean over the colony:

$$\langle s\rangle = \lim_{\langle N\rangle\to\infty}\langle\frac{1}{N}\sum_{i=1}^{N} s_i\rangle = s_b e^{\mu t}\lim_{\langle N\rangle\to\infty}\langle\frac{1}{N}\rangle, \tag{15}$$

converging to a finite value as we have shown for the *adder* [2]. Later, we will discuss deeper why this is not the case for the case of the *timer*. If $\langle s\rangle$ is finite, we can consider that each cell size s_i can be written in terms of the mean cell size $\langle s\rangle$ and each particular fluctuation around this mean ε_i following:

$$s_i = \langle s\rangle + \varepsilon_i. \tag{16}$$

This means that the total biomass can be written as:

$$B = \sum_i^N s_i = N\langle s\rangle + \sum_i^N \varepsilon_i. \tag{17}$$

Thus, the population number is expressed as:

$$N = \frac{B}{\langle s\rangle} + \frac{\sum_i^N \varepsilon_i}{\langle s\rangle}. \tag{18}$$

Estimating the moments of (18), we use $\langle\sum_i^N \varepsilon_i\rangle = 0$ and for the second moment:

$$\langle\sum_i^N \varepsilon_i \sum_j^N \varepsilon_j\rangle = \langle\sum_i^N \varepsilon_i^2\rangle \approx \langle N\rangle \mathrm{var}(\varepsilon). \tag{19}$$

with the definition $\mathrm{var}(\varepsilon) = \mathrm{var}(s) < \infty$, being the variance of the cell size distribution. Finally, the moments of (18) present the asymptotic behavior:

$$\begin{aligned}\langle N\rangle &\xrightarrow{\langle N\rangle\to\infty} \frac{B}{\langle s\rangle}\\ \langle N^2\rangle &\xrightarrow{\langle N\rangle\to\infty} \frac{B^2}{\langle s\rangle^2} + \langle N\rangle\frac{\mathrm{var}(s)}{\langle s\rangle^2},\end{aligned} \tag{20}$$

which can be used to estimate the asymptotic behavior of CV^2_N:

$$CV^2_N = \frac{\mathrm{var}(N)}{\langle N\rangle^2} \xrightarrow{\langle N\rangle\to\infty} \frac{1}{\langle N\rangle}CV^2_s. \tag{21}$$

For the particular case of the *timer* division, when N is a geometric-distributed variable with $\langle N\rangle = \exp(\mu t)$, it is possible to show that:

$$\langle\frac{1}{N}\rangle = \frac{\mu t e^{-\mu t}}{1 - e^{-\mu t}}, \tag{22}$$

therefore, the expected value of the arithmetic average of the size over the colony is given by:

$$\langle\frac{1}{N}\sum_i^N s_i\rangle = \frac{\mu t}{1 - e^{-\mu t}} \xrightarrow{t\gg\mu^{-1}} \mu t, \tag{23}$$

In fact, the fluctuations of this arithmetic average grow even faster. With similar approach using the properties of the geometric distributions, the squared coefficient of variation has asymptotic behavior:

$$CV^2\left(\frac{1}{N}\sum_i^N s_i\right) \xrightarrow{t\gg\mu^{-1}} \frac{e^{\mu t}}{(\mu t)^2}. \tag{24}$$

This shows that, for the *timer* division, the arithmetic mean of the cell sizes over the colony does not converge to $\langle s\rangle$.

To overcome this apparent paradox, in [3] and [2], we show that the arithmetic mean of the cell size, from experiments, should be estimated without considering the cell lineage. In other words, consider a large amount of colonies C, each of them having a label $c \in \{1, \cdots, N_c\}$. If each colony has a number N_c of individuals, each of them having sizes $(s_i)_c$ with $i \in \{1, \cdots, N_c\}$, the cell size average that converges to $\langle s^\alpha\rangle$ with $\alpha > 0$ defining the moment of the size (with $\alpha = 1$ we are calculating the mean) corresponds to:

$$\langle s^\alpha\rangle = \lim_{C\to\infty}\frac{\sum_{c=1}^C\sum_{i=1}^{N_c}(s_i^\alpha)_c}{\sum_{c=1}^C N_c}, \tag{25}$$

which we verified from simulations, that matches with the theoretical values in [3]. In [2], we show that for the *adder* this metric

(25) and the arithmetic mean (15) converge to the same value using numerical methods. For the case of *timer* division, the difference between them is higher due to the properties of the division strategy.

Simulating the effects of the capsule shape on the cell allometry

In the main article, we observed how the allometric exponent, that is, the exponent that relates other dimensions and the cell length, is higher than 1. To quantify directly how this non-linearity arises, we study the dimension of different capsule-shapes. The first hypothesis to test was that the non-linearity is given by the capsule shape approximation.

Effects of the capsule shape:

Figure 1A shows the scaling of these cell dimensions as length increases. Each data point was generated following these steps:

a) We consider cell growing exponentially been picked with a random cycle time τ taht follows an uniform distribution in the interval $(0, 1)$.

b) The cell length was estimated from τ and the cell length L_b at the beginning of the cycle. This L_b is picked up from a gamma distribution with mean $\langle L_b \rangle = 1.7$ and $CV^2_{L_b} = 0.015$ with similar moments to the experimentally observed. If the growth rate $\mu = \ln(2)$ is set such as the length is doubled after 1 unit of time, the cell length L follows:

$$L = L_b e^{\mu\tau}; \quad L_b \sim \text{gamma}; \quad \tau \sim U(0, 1). \tag{26}$$

c) The cell width w was an independent random variable with normal distribution with mean $\langle w \rangle = 1$ and standard deviation $\sigma_w = 0.1$.

d) From the selected w and L, the other cell dimensions were estimated using the formulas:

$$\begin{aligned} A &= \pi L w; \quad V = \pi(L - w)\left(\frac{w}{2}\right)^2 + \frac{3}{4}\pi\left(\frac{w}{2}\right)^3 \\ A_p &= w(L - w) + \pi\left(\frac{w}{2}\right)^2. \end{aligned} \tag{27}$$

Figure 1A shows the allometry of capsule shaped-shaped cells. Since the surface area A is linear with L, we found an exponent of 1. However, both, the projected area and the volume show a non-linearity. We can also observe how the size noise between length, area and volume are clearly different and approximately constant over the cell cycle.

Effects of the cell width increasing:

Figure 1B shows the scaling cell dimensions as function of the length considering that the width increases as function of the cell length. Each data point was generated following these steps:

a) Cell length L was generated randomly using identical procedures as the previous case (numeral a) and b)).

b) Given L, the cell width w was a random variable following:

$$w = \alpha L^{\beta} + \eta; \quad \eta \sim N(0, \sigma_w), \tag{28}$$

with $\alpha = 0.97$ and $\beta = 0.06$. η is the noise in w following a normal distribution with mean 0 and standard deviation $\sigma_w = 0.1$

c) From the selected w and L, the other cell dimensions were estimated using (27).

Figure 1B shows the allometry of the cells with this non-constant width. Overall, the allometric exponents increase compared to the obtained with the constant width simulation. However, the cell size noise does not change very much compared to the observed and is almost constant over the cell cycle.

Effects of the decreasing of the noise width:

In the main text we observe that the cell volume noise decreases over the cell cycle. Similarly, we notice that the width variability of the cells also decreases over the cell cycle. We hypothesize that this reduction in width variability could be the cause of the reduction in volume noise. To test this hypothesis, we simulate the size statistics of the cells with a decreasing width variability over the cycle. We generate each data point by following these steps:

a) Cell length L was generated randomly using identical procedures as the previous case (numeral a) and b)).

b) Given L, the cell width w was a random variable following:

$$w = \alpha L^{\beta} + \eta; \quad \eta \sim N(0, \sigma_w), \tag{29}$$

with $\alpha = 0.97$ and $\beta = 0.06$. η is the noise in w following a normal distribution with mean 0 and standard deviation depends on the length following: $\sigma_w = 0.13 - 0.015L$.

c) From the selected w and L, the other cell dimensions were estimated using (27).

Figure 1C shows the allometry of the cells with this non-constant width and width noise decreasing over the cell cycle. While the noise in length and surface area show almost not variability over the cell cycle, we observe the decreasing on the volume noise. Furthermore, the allometric exponents do not change very much compared to the obtained exponents with only the width increasing .

Simulation details for the *timer* division

As main parameter of the simulation using the timer division strategy we use the cell cycle noise $CV^2_{\tau_d}$. Under the assumption that the mean cell cycle is $\langle \tau_d \rangle$, τ_d follows a gamma distribution:

$$gamma(\tau_d; k, \theta) = \frac{1}{\Gamma(k_\tau)\theta_\tau}\left(\frac{\tau_d}{\theta_\tau}\right)^{k-1} e^{-\frac{\tau_d}{\theta_\tau}}, \tag{30}$$

where the parameters can be estimated from $CV^2_{\tau_d}$ using:

$$k_\tau = \frac{1}{CV^2_{\tau_d}}, \quad \theta_\tau = \langle \tau_d \rangle CV^2_{\tau_d}. \tag{31}$$

In algorithm 1, we present the pseudo-code for the simulation of the colony proliferation for cells dividing using the timer strategy with cycle duration drawn from gamma distribution with parameters explained in (31).

We used an oriented based simulation to model the cell division process. The main idea is illustrated in algorithm 1. A colony is represented by an array that initially contains one element. This element is the time to division of the first cell, which is drawn

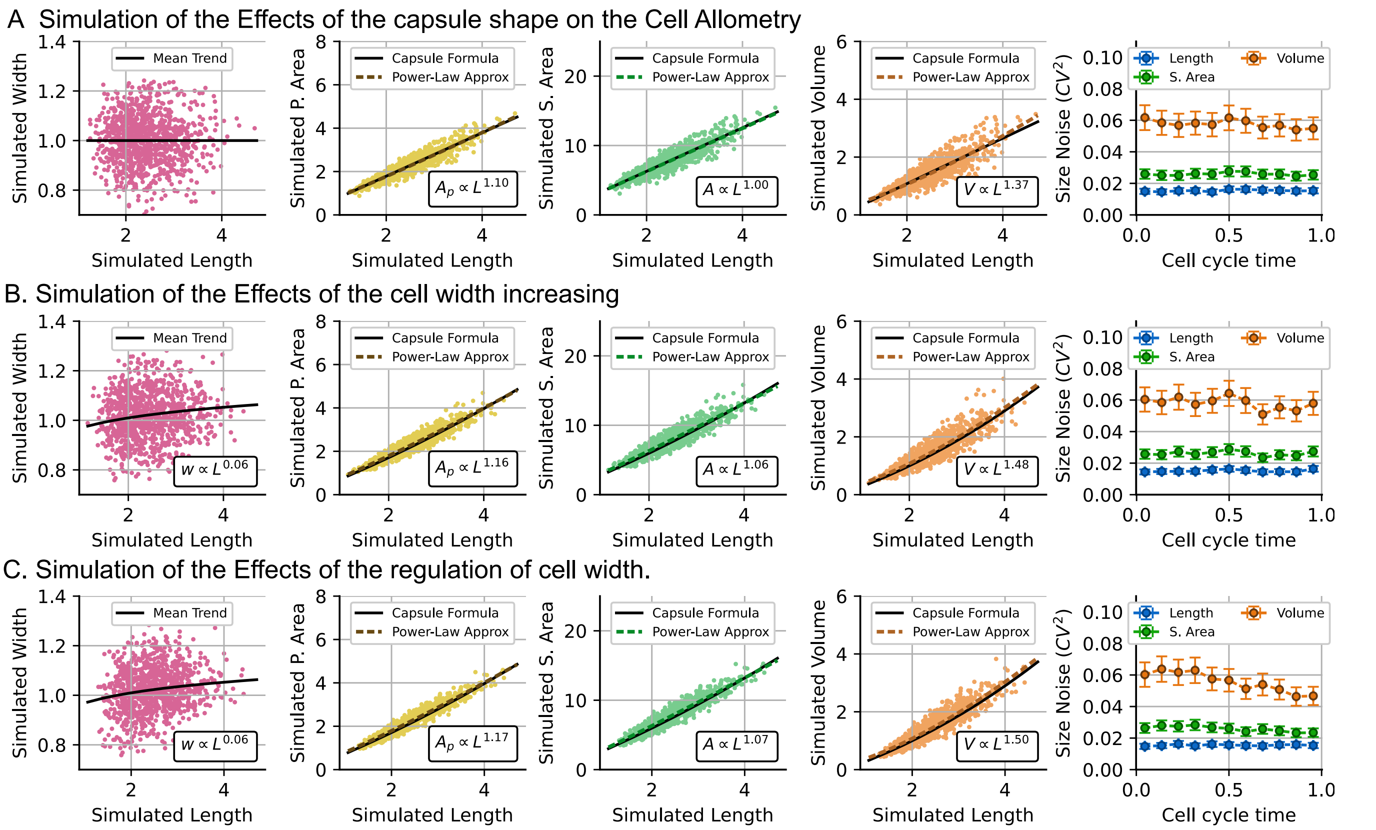

Figure 1: **Origin of the observed trends in cell size statistics A.** Allometry of cell capsules and the size noise over the cell cycle. **B.** Scaling exponents considering a cell width increasing with the cell length. **C.** Scaling exponents considering a cell width increasing with the cell length and noise in the width decreasing with the length. While the plot shows 1 000 points, the statistics cas done with 10 000 data points.

from the gamma distribution (30). The simulation advances the time until this event occurs and then adds a new element to the array, corresponding to a new cell. The time to division of both the parent and the daughter cells are reset to new random variables from the same distribution. If the array has more than one cell, the next division event is determined by the minimum time to division among all cells. The remaining times to division of the other cells are reduced by the elapsed time accordingly. Always there is a division, one new cell is added to the array. Cell simulation stops when the next time to division is scheduled to happen after the maximum time to division. This time can be either the final time or a time for data sampling. The algorithm can resume after this stop.

Simulation details for the *adder* division

For this division strategy, the main parameters are the mean added size during division $\langle\Delta\rangle$, the noise in added size CV^2_Δ, the growth rate μ and the size of the progenitor size s_b.

The main idea of the simulation is presented in algorithm 2. Observe that one of the inputs of the simulation is the progenitor size s_b. As explained in the main text, this is a random variable. Given the variability on the progenitor cell size $CV^2_{s_b}$, we assume that the mean progenitor size is satisfies $\langle s_b\rangle = \langle\Delta\rangle = 1$. Hence, s_b is drawn from a gamma distribution with parameters estimated from $\langle s_b\rangle$ and $CV^2_{s_b}$ from similar formulas to (31).

In the *adder* division strategy, the added size during a cell cycle is a random independent variable. In our simulation, we consider Δ to be a gamma distributed variable similarly as (30):

$$gamma(\Delta; k_\Delta, \theta_\Delta) = \frac{1}{\Gamma(k_\Delta)\theta_\Delta}\left(\frac{\Delta}{\theta_\Delta}\right)^{k-1} e^{-\frac{\Delta}{\theta_\Delta}}, \qquad (32)$$

where the parameters can be estimated from CV^2_Δ using:

$$k_\Delta \frac{1}{CV^2_\Delta}, \quad \theta_\Delta = \langle\Delta\rangle CV^2_\Delta. \qquad (33)$$

Let s_b be the size at the beginning of the cycle and Δ be the added size for that cycle. Thus, the size to division is given by $s_d = s_b + \Delta$.

If we assume that the cell size is growing exponentially over time $s_d = s_b e^{\mu\tau_d}$, therefore, the cell cycle time τ_d is given by:

$$\tau_d = \frac{1}{\mu}\ln\left(\frac{s_b + \Delta}{s_b}\right). \qquad (34)$$

Other difference with the simulation for the *timer* division is that each cell size has to be tracked. To do that, we needed an additional array that we called *sizes* in algorithm 2. Each element is the cell size at division to the next cell splitting. Again, after each division, a new element is added to the arrays *times* and *sizes* corresponding to one cell.

Experimental details

Biological preparation and medium

In this study, the bacterial strain *Corynebacterium glutamicum* ATCC 13032 was used. *C. glutamicum* was cultivated in CGXII

Data: $CV^2_{\tau_d}$, $\langle\tau_d\rangle$, $t_{max} > 0$
Result: $times = [(\tau_d)_1, (\tau_d)_2, \cdots, (\tau_d)_N]$
$(\tau_d)_1 \sim gamma(k_\tau, \theta_\tau)$;
$times = [(\tau_d)_1]$;
$\tau_{min} = (\tau_d)_1$;
$\tau = \tau_{min}$;
if $\tau < t_{max}$ **then**
  while $\tau < t_{max}$ **do**
    $m = argmin(times)$;
    $\tau_{min} = min(times)$;
    for $i = 0;\ i < length(times);\ i = i + 1$ **do**
      $times[i] = times[i] - \tau_{min}$;
    end
    $times[m] \sim gamma(k_\tau, \theta_\tau)$;
    $N = length(times)$;
    $times[N] \sim gamma(k_\tau, \theta_\tau)$;
    $m = argmin(times)$;
    $\tau_{min} = min(times)$;
    $\tau = \tau + \tau_{min}$;
  end
end

Algorithm 1: Algorithm for the simulation of the proliferation of one colony considering the timer division strategy. Given the size mean cycle duration $\langle\tau_d\rangle$, its variability $CV^2_{\tau_d}$ and the time to end the simulation t_{max}, the algorithm calculates the array *times* corresponding to the time left for next division for all cells in colony. The population corresponds to the number of elements of *times*. $gamma(k_\tau, \theta_\tau)$ corresponds to a gamma distributied random variable with parameters explained in (31).

medium [4]. CGXII medium consists of the following components per liter of demineralized water: 20 g of (NH4)2SO4, 1 g of K2HPO4, 1 g of KH2PO4, 5 g of urea, 13.25 mg of CaCl2·H2O, 0.25 g of MgSO4·7H2O, 10 mg of FeSO4·7H2O, 10 mg of MnSO4·H2O, 0.02 mg of NiCl2·6H2O, 0.313 mg of CuSO4·5H2O, 1 mg of ZnSO4·7H2O, 0.2 mg of biotin and 40 g of D-glucose. The concentration of protocatechuic acid (PCA) was 30 mg/L in the medium. For shake flask cultivation, 42 g/L MOPS buffer was added. The solutions were autoclaved or sterile filtered. Medium was adjusted to a pH of 7., All used chemicals were purchased from Carl Roth.

For preculture of *C. glutamicum*, 10 mL CGXII medium in a 100-mL baffled shake flask were inoculated from a cryostock and cultivated overnight at 30°C on a rotary shaker at 120 rpm. The preculture was transferred from a glycerol inoculated into a 100 mL shake flask with 10 mL working volume and cultivated overnight. The main culture was prepared from the preculture inoculated at an initial optical density (OD600) of 0.05. After reaching an OD600 0.2, the culture was transferred to a 1 mL syringe and the microfluidic chip was inoculated.

Chip design

For our microfluidic experiment, a microfluidic chip was adapted from Täuber *et al.* [5]. The chip consists on 14 arrays of cultivation chambers (80 µm x 90 µm x 650 nm) arranged in seven blocks. There are around 400 µm between each array block to separate the zones for a stable flow profile and to test five different stress duration in one experiment. The supply channels have

Data: CV^2_Δ, $\langle\Delta\rangle$, $t_{max} > 0, s_b$
Result: $\{times = [(\tau_d)_1, (\tau_d)_2, \cdots, (\tau_d)_N]$,
$sizes = [(s_d)_1, (s_d)_2, \cdots, (s_d)_N]\}$
$\Delta \sim gamma(k_\Delta, \theta_\Delta)$;
$(\tau_d)_1 = \frac{1}{\mu}\ln\left(\frac{s_b+\Delta}{s_b}\right)$;
$(s_d)_1 = s_b + \Delta$;
$times = [(\tau_d)_1]$;
$sizes = [(s_d)_1]$;
$\tau_{min} = (\tau_d)_1$;
$\tau = \tau_{min}$;
if $\tau < t_{max}$ **then**
  while $\tau < t_{max}$ **do**
    $m = argmin(times)$;
    $\tau_{min} = min(times)$;
    for $i = 0;\ i < length(times);\ i = i + 1$ **do**
      $times[i] = times[i] - \tau_{min}$;
    end
    $s_b = sizes[m]/2$;
    $\Delta \sim gamma(k_\Delta, \theta_\Delta)$;
    $times[m] = \frac{1}{\mu}\ln\left(\frac{s_b+\Delta}{s_b}\right)$;
    $sizes[m] = s_b + \Delta$;
    $N = length(times)$;
    $\Delta \sim gamma(k_\Delta, \theta_\Delta)$;
    $times[N] = \frac{1}{\mu}\ln\left(\frac{s_b+\Delta}{s_b}\right)$;
    $sizes[N] = s_b + \Delta$;
    $m = argmin(times)$;
    $\tau_{min} = min(times)$;
    $\tau = \tau + \tau_{min}$;
  end
end

Algorithm 2: Algorithm for the simulation of the proliferation of one colony considering the adder division strategy. Given added size $\langle\Delta\rangle$, its variability CV^2_Δ and the time to end the simulation t_{max}, the algorithm calculates the array *times* corresponding to the time left for next division for all cells in colony and the array *sizes* which is the array of size to division. The population corresponds to the number of elements of *times*. $gamma(k_\Delta, \theta_\Delta)$ corresponds to a gamma distributied random variable with parameters explained in (31).

a height of 10 µm and a width of 100 µm (See Figure 2 in main text.)

Chip fabrication

The microfluidic chips were fabricated using the soft lithography technique. In this process, PDMS was deposited on a silicon wafer in a ratio 10:1 between current agent and linker (Sylgard 184 Silicone Elastomer, Dow Corning Corporation, USA). After degassing the PDMS, it was baked at 80°C for 2 h. Then, the PDMS chips were cut out from the wafer and the inlets and outlets were punched (Reusable Biopsy Punch, 0.75 mm, WPI, USA). The PDMS chip and a glass slide (D 263 T eco, 39.5×34.5×0.175 mm, Schott, Germany) were cleaned three times with isopropanol and both parts were activated by O2 plasma cleaner (Femto Plasma Cleaner, Diener Electronics, Ebhausen, Germany) and assembled.

Microfluidic single-cell cultivation

In a randomized process, the cultivation chambers were loaded with 1-5 cells. When sufficient cultivation chambers were loaded, the flow of the cell suspension was stopped and the flow of medium was started using high precision pressure pumps (Line-up series, Fluigent, Jena, Germany). A pressure of 180 and 20 mbar was set to initiate medium flow. The detailed flow profile was published in Täuber et al. [6].

Live cell imaging

Live cell imaging was performed using an automated inverted microscope (Nikon Eclipse Ti2, Nikon, Germany). The microscope is placed in a cage incubator for optimal temperature control at 30 ℃ (Cage Incubator, OKO Touch, Okolab S.R.L., Italy). The microfluidic chip was attached to a homemade chip holder. To study the bacterial cells, a 100× oil objective (CFI P-Apo DM Lambda 100× Oil, Nikon GmbH, Germany), a DS-Qi2 camera (Nikon GmbH, Germany), and an automatic focusing system (Nikon PFS, Nikon GmbH, Germany) were used to prevent thermal drift during cultivation. 150 cultivation chambers were manually selected for each experiment using NIS Elements software (Nikon NIS Elements AR software package, Nikon GmbH, Germany). Phase-contrast images of each position were taken every 10 min with an exposure time of 50 ms and a lamp intensity of 10%.

References

[1] Nieto, C., Vargas-García, C., Pedraza, J. M., and Singh, A. (2022). Modeling cell size control under dynamic environments. IFAC-PapersOnLine, 55(40), 133-138.

[2] Nieto, C., Vargas-García, C., Pedraza, J. M., and Singh, A. (2022, December). Cell size control shapes fluctuations in colony population. In 2022 IEEE 61st Conference on Decision and Control (CDC) (pp. 3219-3224). IEEE.

[3] Totis, N., Nieto, C., Küper, A., Vargas-García, C., Singh, A., and Waldherr, S. (2020). A population-based approach to study the effects of growth and division rates on the dynamics of cell size statistics. IEEE Control Systems Letters, 5(2), 725-730.

[4] Unthan, S., Grünberger, A., van Ooyen, J., Gätgens, J., Heinrich, J., Paczia, N., et al. (2014). Beyond growth rate 0.6: What drives Corynebacterium glutamicum to higher growth rates in defined medium. Biotechnol. Bioeng. 2, 359–371. doi: 10.1002/bit.25103.

[5] Täuber, S., Golze, C., Ho, P., Lieres, E., and von, Grünberger, A. (2020). dMSCC: a microfluidic platform for microbial single-cell cultivation of Corynebacterium glutamicum under dynamic environmental medium conditions. Lab Chip 20, 4442–4455. doi: 10.1039/D0LC00711K.

[6] Täuber S, Blöbaum L, Wendisch VF and Grünberger A (2021) Growth Response and Recovery of Corynebacterium glutamicum Colonies on Single-Cell Level Upon Defined pH Stress Pulses. Front. Microbiol. 12:711893. doi: 10.3389/fmicb.2021.711893.
